# Challenges with organization, discoverability and access in Canadian open health data repositories

**DOI:** 10.29173/jchla29457

**Published:** 2021-04-02

**Authors:** Gail M. Thornton, Ali Shiri

**Affiliations:** 1 School of Library and Information Studies, University of Alberta, Edmonton, AB; 2 Department of Mechanical Engineering, University of Alberta, Edmonton, AB

## Abstract

**Introduction:**

Open health data provides healthcare professionals, biomedical researchers and the general public with access to health data which has the potential to improve healthcare delivery and policy. The challenge is to create and implement appropriate metadata, or structured data about the data, to ensure that data are easy to discover, access and re-use. The goal of this study is to identify, evaluate and compare Canadian open health data repositories for their searching, browsing and navigation functionalities, the richness of their metadata description practices, and their metadata-based filtering mechanisms.

**Methods:**

Metadata-based search and browsing was evaluated in addition to the number and nature of metadata elements. Six Canadian open health data repositories across national, provincial and institutional levels were evaluated. Data collected using verbatim text recording was evaluated using an analytical framework based on the 2019 Dataverse North Metadata Best Practices guide and 2019 Data Citation Implementation Project roadmap.

**Results:**

All repositories required filtering to access “open health data.” All repositories included ‘subject’ facets for filtering, and ‘title’ and ‘description’ on the Results List. Use case evaluations suggest improvements including advanced search, health-specific search terms, records for all repositories, and links to related publications.

**Discussion:**

Consistent use of ‘title’ and ‘description’ suggests that an interoperable interface is possible. Inconsistencies in records indicate the need for explicit, easy to find mechanisms to access metadata in repositories. The analytical framework represents first draft guidelines for metadata creation and implementation to improve organization, discoverability, and access to Canadian open health data.

## Introduction

Evidence-based medicine depends on health data. Open health data gives healthcare professionals, biomedical researchers, and the general public access to health data that can improve healthcare delivery and affect healthcare policy [1]. The use of metadata (structured data about the data) to assist users with discovering and accessing open health data is not well studied or understood. Interestingly, Dixit et al. (2018) found the most significant issue with usability of a dataset was incomplete, inconsistent, and poor-quality metadata [2]. The impact of open health data is impeded by poor metadata practices when such practices make the datasets difficult to discover and access for the various interested audiences.

Open access initiatives (e.g. Budapest Open Access Initiative [3]) and open government initiatives (e.g. Government of Canada [4]) have made open health data more available. However, data providers have been given limited guidance regarding what information to consistently include in the records to ensure that the data is discoverable and usable [5, 6]. Metadata are essential for searching, browsing, and re-using data [6]. The gap in current research and practice arises from the fact that making the data available has been emphasized over making the data easy to find.

Most of the research on open health data repositories has occurred in the last five years, which indicates its importance as an emerging field of study. Evaluations of metadata in open health data repositories focussed on adherence to the Dublin Core (DC) metadata standard [7,8] and the Open Archive Initiative-Protocol for Metadata Harvesting (OAI-PMH) interoperability standard [9], and metadata for datasets in the repositories [2,10-12]. The DC metadata standard is a simple and effective set of elements to describe various networked resources [13]. The DC metadata standard has 15 elements [14]: contributor, coverage, creator, date, description, format, identifier, language, publisher, relation, rights, source, subject, title, and type. The OAI-PMH interoperability standard allows various search engines to harvest the data from repositories; thereby enabling users to find relevant information from various sources [9].

In addition to considering DC and OAI-PMH standards, this research will consider previous approaches used to evaluate the use of metadata in searching, browsing, and navigational functionalities to ensure discoverability and access. Ismond and Shiri (2007) evaluated the search and browsing functionalities of six medical digital libraries in addition to the metadata on results and records, including recording the number of DC elements [15]. Farnel and Shiri (2014) [16] examined four research data repositories using analytical frameworks based on the DC metadata standard and National Information Standards Organization (NISO) principles of good metadata [17], which include the use of metadata (e.g. DC), interoperability (e.g. OAI-PMH), licensing, versioning, and identifiers. Schauppenlehner and Muhar (2018) performed an analysis of search functionalities and qualitative text analysis of metadata for two open data repositories [18]. Our approach to the evaluation of metadata in Canadian open health data repositories was to develop an analytical framework that incorporates DC and NISO standards. Inconsistencies identified across the open health data repositories will provide an opportunity to evaluate how end users will be impacted by considering use cases [19] for the three users of interest: healthcare professionals, biomedical researchers and the general public.

Despite some evaluations of the Government of Canada open data repository in international studies of principles of open data [1] and usability of open data repositories [20], Canadian open health data repositories and their use of metadata have been largely overlooked. The Canadian Institute for Health Information (CIHI) is integral to Canada’s position as a global leader in administrative health data science [21]. CIHI is a not-for-profit, independent organization providing information on the health of Canadians and Canadian health systems [22]. With respect to open data repositories in Canada, the Federated Research Data Repository (FRDR) is a collaboration between the Canadian Association of Research Libraries (CARL) / Portage and Compute Canada [23]. As of January 2019, FRDR had forty-four collaborating repositories [24], which include federal, provincial, and municipal government repositories and institutional repositories. FRDR uses the fifteen element DC metadata standard. Even though limited attention has been paid to Canadian open health data repositories, the current research will build on Canada’s strengths as a global leader in administrative health data [21] and an innovator in open data [23].

As detailed in the preceding, the gap in current research and practice arises from the fact that making the data available has been emphasized over making the data easy to find. Datasets have great potential for re-use because users other than the original contributors could perform further analysis on a dataset or combine a dataset with other datasets from within or outside the same repository. The unmet need is to examine the current state of Canadian open health data repositories where standardizing metadata on records would permit easier discovery, access, and re-use of data by various user groups.

The goal of this mixed-methods study is to identify, evaluate and compare Canadian open health data repositories for their searching, browsing and navigation functionalities, the richness of their metadata description practices, and their metadata-based filtering mechanisms. In addition, the consistency of the metadata elements will be contrasted across various Canadian open health data repositories, including governmental and institutional repositories. Further, the analysis will consider the adherence to appropriate standards for metadata and interoperability (ability to interact with other systems). An analytical framework will be developed and applied to the analysis of metadata on records. Inconsistencies will be evaluated based on use cases for the multiple users identified for open health data repositories: healthcare professionals, biomedical researchers and the general public. This research is framed by the following four questions:

- What are the metadata-based searching and browsing functionalities of Canadian open health data repositories?
- How many metadata elements and what metadata elements are available in Canadian open health data repositories?
- What metadata elements are similar and what metadata elements are different across Canadian open health data repositories? • Which Canadian open health data repositories follow metadata standards and interoperability standards?

## Methods

This research follows the pragmatic theoretical framework where the research problem is the most important consideration and all methods required to address the research problem can be applied [25, 26]. This exploratory study is part of the general area of research problems addressing the challenges with organization, discoverability, and access in digital and open resources. The exploratory nature of this study required a qualitative approach; however, evaluation of the number of metadata elements used for the metadata-based functionalities required a quantitative approach. The combined qualitative and quantitative approach is considered a mixed-methods approach [25, 26].

The methods used are based on previous evaluations of digital libraries and data repositories [15, 16, 18]. The current study represents a significant advancement from the pre-tested methods presented previously by Thornton and Shiri (2019) [27] by evaluating an additional repository, developing an analytical framework, and evaluating use cases. The approach could be considered content analysis or text analysis of the records. However, the functionalities of searching, browsing and navigation are considered, which extends the evaluation beyond just the content of the record, to how the information is presented to the user to allow discoverability and access. This is not a usability study but attempts to provide some evaluation of how a user would interact with the metadata. Wu et al. (2019) considered use cases to build their requirements and recommendations for data discovery in data repositories and recommended that data repositories should strive for consistency with other repositories for improved usability and functionality [19]. The current study evaluates the consistency between Canadian open health data repositories.

### Developing the Analytical Framework

Our approach to the evaluation of metadata in Canadian open health data repositories was to develop an analytical framework that incorporates DC and NISO standards. The analytical framework that was applied to Canadian open health data repositories was a combination of two frameworks that were published in April 2019. First, the Dataverse North (DVN) Metadata Best Practices guide was produced by the Metadata subgroup of the Dataverse North Working Group on behalf of the CARL [28]. The DVN Metadata Best Practices fall into required, recommended and optional categories. The relevant DC elements are listed in parenthesis. The DVN required metadata are Title (‘title’), Author (‘creator’), Description (‘description’), Subject (‘subject’), Producer (‘publisher’) and Contact including name, affiliation, and email. Second, the Data Citation Roadmap for Scholarly Data Repositories was developed by the Repositories Expert Group of the Data Citation Implementation Project (DCIP), which is an initiative of FORCE11.org and the National Institutes of Health (NIH)-funded BioCADDIE project [29]. The DCIP roadmap addresses metadata for data discovery and citation. The metadata for data citation were Dataset Identifier (‘identifier’), Title (‘title’), Creator (‘creator’), Publisher (‘publisher’), Publication Date (‘date’), Type (‘type’) and Version. The metadata for data discovery were Description (‘description’), Keywords (‘subject’), License (‘license’), Related Publication (‘relation’) and Related Dataset. The analytical framework was developed by combining the DVN guide [28] and DCIP roadmap [29] (Table 1).

**Table 1:** Analytical framework

Analytical Framework with Dublin Core *(DC)* elements	Dataverse North (DVN) Metadata Best Practices guide required metadata	Data Citation Implementation Project (DCIP) roadmap metadata for data discovery and citation
Title *(title)*	Title	Title (citation)
Creator *(creator)*	Author	Creator (citation)
Description *(description)*	Description	Description (discovery)
Subject *(subject)*	Subject	Keywords (discovery)
Publisher *(publisher)*	Producer	Publisher (citation)
Contact Name	Contact Name	
Contact Affiliation	Contact Affiliation	
Contact Email	Contact Email	
Identifier *(identifier)*		Dataset Identifier (citation)
License *(license)*		License (discovery)
Date *(date)*		Publication Date (citation)
Type *(type)*		Type (citation)
Related Publication *(relation)*		Related Publication (discovery)
Version*		Version (citation)
Related Dataset*		Related Dataset (discovery)

Note: Analytical framework developed from Dataverse North (DVN) Metadata Best Practices guide [28] required metadata and Data Citation Implementation Project (DCIP) roadmap [29] metadata for data discovery and citation. * Related Dataset could be a different Version of the dataset or part of a larger dataset [29].

The metadata common to both the DVN required metadata and DCIP metadata for discovery and citation are ‘title,’ ‘creator,’ ‘description,’ ‘subject’ and ‘publisher.’ The metadata unique to the DVN required metadata was Contact including name, affiliation and email. The metadata unique to the DCIP metadata for data discovery and citation are Dataset Identifier (‘identifier’), License (‘license’), Publication Date (‘date’), Type (‘type’), Related Publication (‘relation’), Version and Related Dataset. Related Dataset could be a different Version of the dataset or a part of a larger dataset [29]. The metadata from the different Canadian open health data repositories was analyzed to determine whether or not the metadata in the developed analytical framework (Table 1) was actually present in the repositories.

The analytical framework combining the DVN Metadata Best Practices guide [28] and DCIP roadmap [29] was aligned with DC metadata elements [14] and NISO good metadata principles [17]. Comparing the NISO good metadata principles with the DCIP roadmap suggestions, many matches are observed. The use of identifiers for the dataset and metadata addresses the second and sixth NISO good metadata principles, respectively. Licensing addresses the fourth and Versioning addresses the fifth NISO good metadata principles. Community standards (first principle) and content standards (third principle) are revealed by the selection and encoding of metadata in the repositories. Considering the focus on metadata-based functionalities in our current study, an analytical framework that combines the specific metadata elements in the DVN guide [28] and DCIP roadmap [29] provides a more efficient analytical approach to evaluate these functionalities than using the more general DC and NISO standards.

### Identifying Open Health Data Repositories

Canadian open health data repositories were identified using open data directories. The first directory used was Directory of Open Access Repositories (OpenDOAR) which is a global directory of open access repositories [30]. As of January 16, 2019, the only Canadian repository with datasets under “Health and Medicine” was Summit from Simon Fraser University; however, Summit itself had no datasets under “Health” or “Medicine” [31]. The second directory used to identify Canadian open health data repositories was FRDR which is a collaboration between CARL/Portage and Compute Canada [23]. As of January 2019, FRDR had forty-four collaborating repositories, which include federal government (e.g. Government of Canada), provincial government (e.g. Government of Alberta), municipal government (e.g. City of Edmonton) and institutional (e.g. University of Alberta Libraries Dataverse) repositories [24].

### Selecting Open Health Data Repositories

Before being selected for evaluation, the repositories had to contain more than 1 open health dataset. Purposive sampling was used to examine 1 repository in every category: federal (Government of Canada), provincial (Government of Alberta), municipal (City of Edmonton) and institutional (University of Alberta Libraries Dataverse). Purposive sampling led to identification of other possible repositories through snowball sampling. For example, the Government of Canada repository included some data from CIHI which is not included in FRDR. Also, the Government of Canada repository included provincial data from only 1 province, Alberta. Unfortunately, the repository for Edmonton had only 1 open health dataset. Based on Edmonton’s limited open health data and the interesting inclusion of 1 province’s data in the federal repository, we elected to consider the repository for another province, British Columbia. Likewise, another institutional repository was added, Scholars Portal Dataverse University of British Columbia (UBC).

The selected repositories were Canadian and contained open data, in particular open health datasets. Six repositories were selected for evaluation:

1. Government of Canada Open Government Portal
2. Canadian Institute for Health Information (CIHI)
3. Government of Alberta Open Data Portal
4. British Columbia (BC) Data Catalogue
5. University of Alberta Libraries (UAL) Dataverse
6. Scholars Portal Dataverse University of British Columbia (UBC Dataverse)

### Evaluating Open Health Data Repositories

Open data repositories were evaluated for their searching, browsing and navigation functionalities, the richness of metadata description practices, and their metadata-based filtering mechanisms. This evidence-based approach was taken to assess the discoverability and access of Canadian open health data repositories.

Data collection was performed by considering the following parameters: Facets (Filters), Browsing, Sorting, Metadata on Results List, Metadata on Record. Data were collected by verbatim text recording of these features from the repositories. Also, basic and advanced search options and the default for sorting options were recorded.

Facets (filters) were evaluated because of their importance in faceted navigation where queries can be refined using facets [32]. Browsing, sorting, metadata on Results List, and metadata on Record were all collected using verbatim text recording [15]. Searching and browsing functionalities including filtering were assessed from the perspective of the user [15, 18]. Metadata on Record was evaluated using the newly-developed analytical framework (Table 1).

## Results

Data was collected from the 6 repositories between January 16, 2019 and April 16, 2019 for filtering open health data repositories and between January 16, 2019 and October 30, 2019 for evaluating open health data repositories.

### Filtering Open Health Data Repositories

The 6 repositories required filtering to get to “open health data” (Table 2). The Government of Canada Open Government Portal [33] was filtered using “Portal Type: Open Data,” “Subject: Health and Safety,” and “Resource Type: Dataset,” which retrieved 1196 records [34]. Interestingly, 335 of the 1196 records were from the Province of Alberta, which was the only provincial jurisdiction included in the repository as demonstrated by the filter “Jurisdiction: Provincial (335).” Additionally, 3 of the 1196 records were from CIHI.

**Table 2:** Filter requirements

Filter	Government of Canada Open Government Portal	Canadian Institute for Health Information (CIHI)	Government of Alberta Open Data Portal	British Columbia (BC) Data Catalogue	University of Alberta Libraries (UAL) Dataverse	Scholars Portal Dataverse University of British Columbia (UBC Dataverse)
open	yes		yes	yes		
health	yes		yes	yes	yes	yes
data	yes	yes	yes	yes		

The CIHI “Access Data and Reports” page has the following filters: primary theme, geography, content format, published date [35]. To separate data from reports, no filter was provided; however, if the only data formats in the “Content format” were selected (XLSX, XLS, and ZIP), then 230 records remained.

Filtering the Government of Alberta Open Data Portal [36] using “Topic: Health and Wellness” resulted in the user being re-directed to the “All Resources” page from the “Open Data” page. An extra step of filtering “Information Type: opendata” was required to get the 358 records [37]. The BC Data Catalogue [38] was filtered using “Sectors: Health and Safety,” “Dataset types: Datasets” and “Download permission: Public” to retrieve 66 datasets [39].

Filtering UAL Dataverse [40] for “Datasets” and “Subject: Medicine, Health and Life Sciences” retrieved 55 records [41]. Filtering UBC Dataverse [42] for “Datasets” and “Subject: Medicine, Health and Life Sciences” retrieved 49 records [43].

For each of these repositories, some filtering was required to get “open health data” (Table 2). For the government repositories, all 3 filters were required (Table 2). CIHI, being a health resource, only required filtering for data (Table 2). UAL and UBC Dataverses, being data resources, only required filtering for health (Table 2). With respect to “open,” CIHI and the Dataverses may have some permission criteria.

### Evaluating Open Health Data Repositories

The 6 open health data repositories have basic search; however, only the UAL and UBC Dataverses have advanced search functionality. The browsing options are only the Results List for 5 of the repositories: Government of Canada, Government of Alberta, BC Data Catalogue, UAL Dataverse, UBC Dataverse. The remaining repository CIHI offers 3 “Frequently accessed,” 3 “Recently released” and 20 “Themes” for browsing their repository. The 20 health-specific “Themes” included “Access and Wait Times,” “Children and Youth,” “Community Care,” “Emergency Care,” “First Nations, Inuit and Métis,” “Health Inequality,” “Health Spending,” “Health System Performance,” “Health Workforce,” “Hospital Care,” “International Comparisons,” “Mental Health and Addictions,” “Organ and Joint Replacements,” “Patient Experience,” “Patient Outcomes,” “Pharmaceuticals,” “Population Health,” “Quality and Safety,” “Residential Care,” and “Seniors and Aging.”

The number of facets for filtering varied from a minimum of 4 for CIHI and a maximum of 9 for Government of Canada (Table 3). All 6 repositories offer a ‘subject’ facet. Three repositories used the term subject for filtering (Government of Canada, UAL Dataverse and UBC Dataverse). The subjects were not Subject Headings like Library of Congress Subject Headings (LCSH) or Medical Subject Headings (MeSH) but were from encoding schemes/controlled lists. In the Keyword field, Dataverse allows identification of keywords from LCSH and MeSH controlled vocabularies. The 2 provincial government repositories do not use the term subject but rather “Topics” (Alberta) or “Sectors” (BC). CIHI uses “Primary theme” which are the same as the “Themes” available for browsing on the “Access Data and Reports” page. Four repositories filter using ‘format,’ 4 filter using ‘date,’ and 3 filter using ‘type’ (Table 3). Five of the repositories provided either ‘publisher’ (3) or ‘creator’ (2) as a facet (Table 3).

**Table 3:** Facets (filters)

Government of Canada Open Government Portal	Canadian Institute for Health Information (CIHI)	Government of Alberta Open Data Portal	British Columbia (BC) Data Catalogue	University of Alberta Libraries (UAL) Dataverse	Scholars Portal Dataverse University of British Columbia (UBC Dataverse)
Portal Type Collection Type Jurisdiction Organization^p^ Keywords Subject^s^ Format^f^ Resource Type^t^ Maintenance and update frequency	Primary theme^s^ Geography Content format^f^ Published date^d^	Information Type Topics^s^ Publisher^p^ Formats^f^ Audience Publication Type^t^ Date Added to Catalogue^d^	License Sectors^s^ Dataset types^t^ Formats^f^ Organizations^p^ Download permission	Metadata Source Publication Year^d^ Author Name^c^ Subject^s^ Keyword Term Deposit Date	Publication Year^d^ Author Name^c^ Subject^s^ Keyword Term Deposit Date

Common Dublin Core elements:

^s^subject = 6; ^f^format = 4; ^d^date = 4; ^t^type = 3; ^p^publisher = 3; ^c^creator = 2

The maximum sorting options were 10 for Government of Alberta and the minimum were 2 for CIHI (Table 4). Excluding the Dataverses, all of the repositories included relevance ranking as a sorting option for the Results List. While Government of Canada and CIHI had relevance sorting as the default, the remaining repositories used a descending date-based default sorting (Table 4).

**Table 4:** Sorting

Government of Canada Open Government Portal	Canadian Institute for Health Information (CIHI)	Government of Alberta Open Data Portal	British Columbia (BC) Data Catalogue	University of Alberta Libraries (UAL) Dataverse	Scholars Portal Dataverse University of British Columbia (UBC Dataverse)
Relevance^d^ Name ascending Name descending Last modified	Relevance^d^ Date	Date last updated ascending Date last updated descending^d^	Relevance Popular Name Ascending	Name (A-Z) Name (Z-A) Newest^d^ Oldest	Name (A-Z) Name (Z-A) Newest d Oldest
		Date added to portal ascending Date added to portal descending Publication date ascending Publication date descending Title ascending Title descending Last Modified Relevance	Name Descending Published Date^d^ Last Modified		

^d^Indicates default

All 6 open health data repositories included the ‘title’ and ‘description’ in the metadata on the Results List (Table 5). Excluding CIHI, the title in the Results List was hyperlinked to the record. For CIHI, the title in the Results List was hyperlinked to the file download. Notably, ‘title’ and ‘description’ were consistent across all repositories in the Results List which suggests that an interoperable interface could be provided to search across all repositories.

**Table 5:** Metadata on Results List

Government of Canada Open Government Portal	Canadian Institute for Health Information (CIHI)	Government of Alberta Open Data Portal	British Columbia (BC) Data Catalogue	University of Alberta Libraries (UAL) Dataverse	Scholars Portal Dataverse University of British Columbia (UBC Dataverse)
Title^t^ (link to record) Jurisdiction Description^d^ Organization Issued by (Jurisdiction) Resource Formats	Title^t^ (link to download) Date Description^d^ Tags (including) Primary theme Geography Content format	Title^t^ (link to record) Information Type Formats Views Last Modified Description^d^ Tags	Title^t^ (link to record) Dataset types Sectors Formats Description^d^ Record Published	Title^t^ (link to record) Publication Date Local Dataverse Citation Description^d^	Title^t^ (link to record) Publication Date Local Dataverse Citation Description^d^

Common Dublin Core elements: ^t^title = 6; ^d^description = 6

The Government of Alberta repository does not explicitly refer to metadata on the record but it is the repository with the best supporting documentation for the metadata which details encoding schemes, metadata standards and DC correlations [44]. Four repositories explicitly refer to metadata on the record (Table 6): Government of Canada, BC Data Catalogue, UAL Dataverse, UBC Dataverse. The BC Data Catalogue (under “Metadata Information”) refers to published and modified dates for the record and status of the resource. With greater effort to address metadata on the record, Government of Canada (under “Metadata”) and UAL and UBC Dataverses (under “Export Metadata”) provide links to export metadata in different standards (Table 7): 3 for Government of Canada, 4 for UAL Dataverse, 6 for UBC Dataverse. The 2 Dataverses offer metadata export in Schema.org JavaScript Object Notation for Linked Data (JSON-LD) and the Government of Canada offers Data Catalog Vocabulary [DCAT (JSON-LD)]. This suggests that some priority was placed on clarifying, within the record itself, the use of metadata standards and support for interoperability.

**Table 6:** Metadata on Record

Government of Canada Open Government Portal	Canadian Institute for Health Information (CIHI)	Government of Alberta Open Data Portal	British Columbia (BC) Data Catalogue	University of Alberta Libraries (UAL) Dataverse	Scholars Portal Dataverse University of British Columbia (UBC Dataverse)
Title Description Publisher – Current Organization Name Licence Resources Resource Name Resource Type Format Language Links [Access] (button to download) Additional Information Contact Email Keywords Subject Maintenance and Update Frequency Date Published Date Modified Openness Rating About this Record Record Released Record Modified Record ID Metadata Link to JSON format DCAT (JSON-LD) DCAT (XML)	*No record*	Title *Summary Tab* Description Tags Resources Resource name (link to download) [More Information] or [Preview] [Download] (button to download) downloads *Detailed Information Tab* Title and Dataset Information Alternative Title Date Modified Update Frequency Publisher/Creator Information Creator Publisher Subject Information Topic Start Date End Date Spatial Coverage Resource Dates Date Created Date Added to Catalogue Date Issued Date Modified Audience information Language Identifiers Usage/Licence Usage Considerations Licence Contact Contact Name Contact Email *Related Tab* list/link related records	Title Dataset types Sectors Views Published by Licensed under Description Tags Activity Stream Data and Resources Filename (file size) [Explore > Preview or Download] Additional Information Data Quality Lineage Statement More Information Contact Information Name Email Organization Suborganization Access & Security Who can view this dataset? Who can download this dataset? Metadata Information Record Published Record Last Modified Resource Status	Title Version Citation [Cite Dataset] Description Subject Keyword Related Publication *Files Tab* Search bar Number of Files Filename file format, file size, date, downloads [Download] (button to download) *Metadata Tab* [Export Metadata] Dublin Core DDI JSON Schema.org JSON-LD Citation Metadata Dataset Persistent ID Publication Date Title Alternative Title Other ID Author Contact [use email button] Name (Affiliation) Description Subject Keyword Related Publication Producer Production Date Production Place Grant Information Time Period Covered Date of Collection Kind of Data Software	Title Version Citation [Cite Dataset] Description Subject Keyword Related Publication Notes *Files Tab* Search bar Number of Files Filename file format, file size, date, downloads [Download] (button to download) *Metadata Tab* [Export Metadata] Dublin Core DDI DataCite JSON OAI-ORE Schema.org JSON-LD Citation Metadata Dataset Persistent ID Publication Date Title Author Contact [use email button] Name (Affiliation) Description Subject Keyword Topic Classification Related Publication Notes Producer Production Date Production Place Grant Information Distributor Distribution Date Depositor Deposit Date Time Period Covered Kind of Data
				Geospatial Metadata Social Sciences and Humanities Metadata Life Sciences Metadata *Terms Tab* Terms of Use Restricted Files + Terms of Access Guestbook *Versions Tab* Dataset Summary Contributors Published	Geospatial Metadata Social Sciences and Humanities Metadata Life Sciences Metadata *Terms Tab* Terms of Use Guestbook *Versions Tab* Dataset Summary Contributors Published

DCAT = Data Catalog Vocabulary

DDI = Data Documentation Initiative

JSON = JavaScript Object Notation

JSON-LD = JavaScript Object Notation for Linked Data

OAI-ORE = Open Archives Initiative Object Reuse and Exchange

XML = eXtensible Markup Language

**Table 7:** Metadata referred to in Metadata on Record

Government of Canada Open Government Portal	Canadian Institute for Health Information (CIHI)	Government of Alberta Open Data Portal	British Columbia (BC) Data Catalogue	University of Alberta Libraries (UAL) Dataverse	Scholars Portal Dataverse University of British Columbia (UBC Dataverse)
Metadata Link to JSON format DCAT (JSON-LD) DCAT (XML)	*No record*	*No explicit use of term metadata on record*	Metadata Information Record Published Record Last Modified Resource Status	*Metadata Tab* [Export Metadata] Dublin Core DDI JSON Schema.org JSON-LD	*Metadata Tab* [Export Metadata] Dublin Core DDI DataCite JSON OAI-ORE Schema.org JSON-LD

DCAT = Data Catalog Vocabulary

DDI = Data Documentation Initiative

JSON = JavaScript Object Notation

JSON-LD = JavaScript Object Notation for Linked Data

OAI-ORE = Open Archives Initiative Object Reuse and Exchange

XML = eXtensible Markup Language

Comparing metadata on the record to the analytical framework based on the DVN guide and DCIP roadmap (Table 1), ‘creator’ was missing from the records for Government of Canada and BC Data Catalogue but was present in the records for the Government of Alberta as Creator and the 2 Dataverses as Author (Table 8). CIHI did not have a record. Examining the Results List and facets demonstrated that CIHI did not identify a creator in a separate metadata field. The DVN guide suggested that Contact include name, affiliation, and email. While all the repositories with records provided some contact information, only 3 repositories had all 3 suggested in the analytical framework (Table 8).

**Table 8:** Metadata on Record aligned with analytical framework

Government of Canada Open Government Portal	Canadian Institute for Health Information (CIHI)	Government of Alberta Open Data Portal	British Columbia (BC) Data Catalogue	University of Alberta Libraries (UAL) Dataverse	Scholars Portal Dataverse University of British Columbia (UBC Dataverse)
Title^a^ Description^a^ Publisher – Current Organization Name^a^ Licence^a^ Resources Resource Name Resource Type^a^ Format Language Links [Access] (button to download) Additional	*No record*	Title^a^ *Summary Tab* Description^a^ Tags Resources Resource name (link to download) [More Information] or [Preview] [Download] (button to download) downloads *Detailed Information Tab* Title and Dataset Information	Title^a^ Dataset types^a^ Sectors^a^ Views Published by^a^ Licensed under^a^ Description^a^ Tags Activity Stream Data and Resources Filename (file size) [Explore > Preview or Download] Additional Information Data Quality	Title Version Citation [Cite Dataset] Description Subject Keyword Related Publication *Files Tab*^a^ Search bar Number of Files Filename file format, file size, date, downloads	Title Version Citation [Cite Dataset] Description Subject Keyword Related Publication Notes *Files Tab*^a^ Search bar Number of Files Filename file format, file size, date, downloads
Information Contact Email^a^ Keywords Subject^a^ Maintenance and Update Frequency Date Published^a^ Date Modified Openness Rating About this Record Record Released Record Modified^a^ Record ID^a^ Metadata Link to JSON format DCAT (JSON-LD) DCAT (XML)		Alternative Title Date Modified Update Frequency Publisher/Creator Information Creator^a^ Publisher^a^ Subject Information Topic^a^ Start Date End Date Spatial Coverage Resource Dates Date Created Date Added to Catalogue^a^ Date Issued Date Modified^a^ Audience information Language Identifiers^a^ Usage/Licence Usage Considerations Licence^a^ Contact Contact Name^a^ Contact Email^a^ *Related Tab*^a^ list/link related records	Lineage Statement More Information Contact Information Name^a^ Email^a^ Organization^a^ Suborganization Access & Security Who can view this dataset? Who can download this dataset? Metadata Information Record Published^a^ Record Last Modified^a^ Resource Status	[Download] (button to download) *Metadata Tab* [Export Metadata] Dublin Core DDI JSON Schema.org JSON-LD Citation Metadata Dataset Persistent ID^a^ Publication Date^a^ Title^a^ Alternative Title Other ID Author^a^ Contact [use email button]^a^ Name^a^ (Affiliation) a Description^a^ Subject^a^ Keyword Related Publication^a^ Producer^a^ Production Date Production Place Grant Information Time Period Covered Date of Collection Kind of Data^a^	[Download] (button to download) *Metadata Tab* [Export Metadata] Dublin Core DDI DataCite JSON OAI-ORE Schema.org JSON-LD Citation Metadata Dataset Persistent ID^a^ Publication Date^a^ Title^a^ Author^a^ Contact [use email button]^a^ Name^a^ (Affiliation)^a^ Description^a^ Subject^a^ Keyword Topic Classification Related Publication^a^ Notes Producer^a^ Production Date Production Place Grant Information Distributor Distribution Date Depositor Deposit Date
				Software Geospatial Metadata Social Sciences and Humanities Metadata Life Sciences Metadata *Terms Tab*^a^ Terms of Use Restricted Files + Terms of Access Guestbook *Versions Tab*^a^ Dataset Summary Contributors Published	Time Period Covered Kind of Data^a^ Geospatial Metadata Social Sciences and Humanities Metadata Life Sciences Metadata *Terms Tab*^a^ Terms of Use Guestbook *Versions Tab*^a^ Dataset Summary Contributors Published
**Missing:** Creator Contact Name Contact Affiliation Related Publication Related Dataset		**Missing:** Contact Affiliation Type (see Table 3)	**Missing:** Creator Identifier Related Publication Related Dataset		

^a^Indicates analytical framework

DCAT = Data Catalog Vocabulary

DDI = Data Documentation Initiative

JSON = JavaScript Object Notation

JSON-LD = JavaScript Object Notation for Linked Data

OAI-ORE = Open Archives Initiative Object Reuse and Exchange

XML = eXtensible Markup Language

The DCIP roadmap suggested persistent identifiers. Under Dataset Persistent ID, the 2 Dataverses have digital object identifiers (DOIs) that identify the dataset and metadata record. The Government of Alberta (under Identifier) and the Government of Canada (under Record ID) have URLs as identifiers. The BC Data Catalogue does not have a specific metadata field for ‘identifier’ (Table 8). The DCIP suggests that related publications and related datasets be provided as metadata for discovery. The 2 Dataverses have Related Publication metadata fields. The Dataverses allow the publication of more than 1 file in the same record to account for related datasets under the Files Tab. Likewise, different versions of the same dataset are accounted for under the Versions Tab in Dataverse. While the BC Data Catalogue and Government of Canada document versioning, related publications and datasets are not considered (Table 8). Government of Alberta addresses versioning and, also, related publications and datasets under the Related Tab.

## Discussion

Six Canadian open health data repositories across national, provincial, and institutional levels were evaluated in terms of information access and metadata practices. The findings of this study suggest that Canadian open health data repositories offer metadata that match many of the suggested metadata in the analytical framework based on the DVN guide and DCIP roadmap. An important contribution of this research was the merging of the metadata from the DVN guide and DCIP roadmap into one analytical framework which essentially represents a first draft of guidelines and best practices for metadata in Canadian open health data repositories.

Filtering was required to get to open health data in all repositories. ‘Subject’ was consistently used for filtering in all repositories even though these were not subject headings, like LCSH or MeSH. Interestingly, ‘title’ and ‘description’ were consistent across all repositories in the Results list. An interoperable interface could be provided to search across these repositories based on the consistent use of ‘title’ and ‘description’. The interoperable interface suggested is a novel and specific contribution of this research. Although FRDR was used to select repositories from the list of collaborating repositories, metadata for the directory itself is harmonized across different schemas on ‘title’ and ‘author’ for consistency [23]. Four repositories refer to metadata within the record itself which indicates the importance of implementing explicit and easy to find mechanisms to access metadata in data repositories, particularly given the role of metadata for searchability, findability and discoverability of open data.

Wu et al. (2019) considered use cases to build their requirements and recommendations for data discovery in data repositories [19]. Canadian open health data repositories need to serve a broad audience including healthcare professionals, biomedical researchers and the general public. The suggestions below were made relatable by providing use cases for one of these three users.

Effectively no health-specific subject searching or browsing for the government and Dataverse repositories was available because the subject filter was already employed to get to “health.” A member of the general public would be better served with additional searching, browsing and filtering of the more specific narrower terms of health subjects that would exist below the broader term of health. Wu et al. (2019) emphasizes that different users may or may not have a clear search target and that a variety of search, browse and filter options can best accommodate various users with various information needs [19].

While CIHI excels in providing many options for searching and browsing, it does not provide a record or landing page as an intermediate between the Results list and the file download. CIHI takes the user directly to the data from the Results list. The challenge for the users, particularly a member of the general public, is that the limited information on the Results list may not be enough to know if the data file download is even desired. Landing pages or records are important for user interaction with the repository but are also critical to the operation of the repository itself. Starr et al. (2015) stresses the importance of resolving identifiers to landing pages rather than directly to the data because the metadata should be a citable part of the scholarly record, hosted even if the data is no longer available and allows for an access point independent of encodings for the data [45]. The CIHI repository should consider the use of landing pages or metadata records for access, citation and preservation of the metadata independent from the data.

Wu et al. (2019) emphasized connecting the dataset with a person [19]. The creator field was missing from the metadata provided by CIHI, Government of Canada, and BC Data Catalogue. Given that CIHI is a health-specific repository, it is surprising that the creator and/or publisher of the dataset is not a metadata field, even in the Results list or facets, because healthcare professionals often conduct searches looking for research from authors or groups who are known to them. Having the dataset easily linked to the related journal article and vice versa allows for two-way discovery. Fenner et al. (2019) suggested that the dataset refer to the publication and the publication refer to the dataset, thereby “enabling navigation between publication and dataset in both directions” [29]. This appears to be a missed opportunity in the government and CIHI repositories. Healthcare professionals would use this feature in evidence-based practice to get the complete picture from all related information.

Only the Dataverses offered advanced search. The other repositories should consider adding advanced search for improved dataset discovery for users who are already familiar with advanced search in other platforms, which was a recommendation for improved data discovery from Wu et al. (2019) [19]. For example, a biomedical researcher would already be familiar with advanced search features in platforms for conducting literature searches [19]. Biomedical researchers would be interested in compiling data on the same topic from multiple repositories. Assante et al. (2016) recognized that most data repositories supported OAI-PMH interoperability but recommended that they should additionally provide access to their content through schema.org and linked data [46]. Both Wu et al. (2019) [19] and Fenner et al. (2019) [29] suggest using schema.org JSON-LD encoding to ensure that the metadata in the repository is machine-readable by Google Dataset Search. The Dataverse platform supports schema.org [47]. UAL and UBC Dataverses offer to export metadata as schema.org JSON-LD. Wide-ranging discovery and interoperability will help biomedical researchers address rare conditions by compiling small sample datasets into large comprehensive datasets.

This study is not without limitations. The approach Martin et al. (2017) used on United States open health data repositories was not applied to Canadian open health data repositories, i.e. their 99-item coding guide for data quality and 29-item coding guide for usability [8]. Martin et al. (2017) reduced their evaluation to a number or index [8]. The current research was less interested in statistically comparing the repositories and preferred to document the richness of the metadata available to the user to interact with the repository. Given the nascent nature of open health data repositories, the focus was to document the details rather than reduce the details to an index. Having said that, future work could consider the importance of usability testing of open health data repositories to evaluate their ability to serve a wide variety of users including healthcare professionals, biomedical researchers and the general public. Future work could consider metadata curation and dataset discovery beyond metadata features explored in the current study. Additionally, after reviewing metadata evaluation approaches for digital libraries, Tani et al. (2013) indicated that these approaches were successful in identifying potential problems but may also require discipline-specific considerations [48]. By identifying potential problems with existing metadata in Canadian open health repositories, this research contributes to the development of guidelines and best practices. This research did not address the use of DATS (DatA Tag Suite) metadata for datasets [49] which can be used for the discipline-specific case of health sciences [23]. By collecting the metadata on the records for the different repositories, analysis comparing against a different analytical framework could be performed at a later date without having to re-collect the data.

In summary, Canadian open health data repositories offer metadata that match many of the suggested metadata in the analytical framework based on the DVN guide and DCIP roadmap. The developed analytical framework, which merges DVN guide and DCIP roadmap metadata and incorporates DC and NISO standards, could be considered a first draft of guidelines and best practices for metadata in Canadian open health data repositories. The opportunities for improvement include a richer search experience for health-specific subjects beyond the first filter to get to the health data, advanced search functionality for users with advanced search experience in other platforms, inclusion of the creator field to search for known authors, records for all repositories for human and machine access to metadata, and links to related publications for two-way discovery. Another novel contribution of this study was the revelation that all six repositories had ‘title’ and ‘description’ in the Results list which means that an interoperable interface could be designed to take advantage of this existing consistency in Canadian open health data repositories. The metadata on the records indicates the need for explicit, easy to find mechanisms to access metadata in repositories. Communication of identified current practices is a contribution of this work and is a first step towards the guidelines and best practices for developing and implementing metadata for open health data repositories that will pave the way for an interoperable open health data environment. These findings will improve the understanding among researchers, librarians, and data managers of the application of metadata in open health data repositories and the challenges associated with finding and discovering open health data.
